# Animal Models for Parkinson’s Disease Research: Trends in the 2000s

**DOI:** 10.3390/ijms20215402

**Published:** 2019-10-30

**Authors:** Kyohei Kin, Takao Yasuhara, Masahiro Kameda, Isao Date

**Affiliations:** Department of Neurological Surgery, Graduate School of Medicine, Dentistry and Pharmaceutical Sciences, Okayama University, 2-5-1, Shikata-cho, Kita-ku, Okayama-shi, Okayama 700-8558, Japan; tyasu37@cc.okayama-u.ac.jp (T.Y.); mrkameda@gmail.com (M.K.); idate333@md.okayama-u.ac.jp (I.D.)

**Keywords:** animal model, α-synuclein, DJ-1, neurotoxin, Parkin, Parkinson’s disease, pesticide, PINK1, 1-methyl-4-phenyl-1,2,3,6-tetrahydropyridine, 6-hydroxydopamine

## Abstract

Parkinson’s disease (PD) is a chronic and progressive movement disorder and the second most common neurodegenerative disease. Although many studies have been conducted, there is an unmet clinical need to develop new treatments because, currently, only symptomatic therapies are available. To achieve this goal, clarification of the pathology is required. Attempts have been made to emulate human PD and various animal models have been developed over the decades. Neurotoxin models have been commonly used for PD research. Recently, advances in transgenic technology have enabled the development of genetic models that help to identify new approaches in PD research. However, PD animal model trends have not been investigated. Revealing the trends for PD research will be valuable for increasing our understanding of the positive and negative aspects of each model. In this article, we clarified the trends for animal models that were used to research PD in the 2000s, and we discussed each model based on these trends.

## 1. Introduction

Parkinson’s disease (PD) is one of the most common neurodegenerative diseases and known as a chronic and progressive disorder [1]. For the most part, PD occurs sporadically and is assumed to result from a complex interaction between environmental and genetic risk factors [2,3,4,5]. Progressive and selective degeneration of dopamine neurons within the substantia nigra (SN) cause the typical symptoms and an increasing dopamine deficit in the striatal axonal projection area [6]. Because the cause in most PD cases remains unclear, only symptomatic therapies such as pharmacotherapy, stereotaxic neurosurgery, and physiotherapy are available. Although these treatments reduce motor symptoms and improve the patient’s quality of life [7], clinical needs for new therapies to prevent, stop, or slow disease progression are not fully understood. Recently, early diagnosis of PD, which occurs several years before the onset of motor symptoms and shows not only some dopaminergic neuronal damage but also non-motor symptoms, was thought to have important implications for the disease-modifying strategies for PD [6]. For the development of such strategies, biomarkers for the diagnosis of early PD were also required. To meet these needs, research using appropriate animal models was invaluable.

When animal studies are conducted, selection of the animal model is critical because the translatability of the research relies on the use of appropriate animal models to emulate the human condition or pathology. The validity of animal models should be considered from the view of face validity (animal models should possess features of human PD), constructive validity (animal models should have a sound theoretical rationale), and predictive validity (animal models should respond to treatments in a manner that is comparable to clinical treatment) [8,9,10]. Because its precise pathomechanism remains unclear, establishment of a complete animal model would be impossible. However, various types of PD animal models have been developed along with the expanding of our knowledge and advance of scientific techniques, and they are used for various purposes. To mimic the typical motor symptoms of idiopathic PD, neurotoxin has been used for animal studies [11,12]. Genomic models have been generated and used for basic research since human genome mutations that are linked to the familial form of PD were identified [13]. However, the actual trend has never been clearly determined. In this article, we clarify the trends for PD animal model in the 2000s and discuss the models based on these trends.

## 2. Trends in PD Animal Models

**Figure 1:**
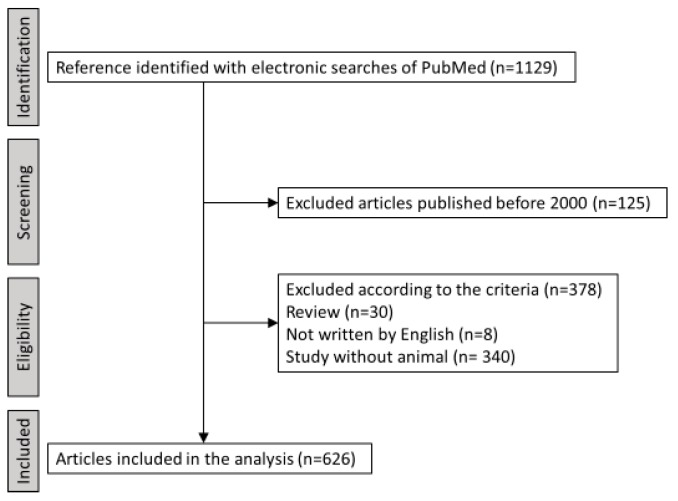
Flow chart for the selection of studies.

To clarify the trends in PD animal models, we identified articles in which the author used animal models in research on PD using electronic searches of PubMed with the terms “Parkinson’s disease” and “animal model” up to and including 18 August 2019. Two investigators (K.K. and Y.T.) reviewed each abstract independently to identify articles that met the inclusion criteria. We included articles that stated the use of any animals as PD animal models and that were written in English. Articles published before 2000 were excluded. The process of selection is shown in Figure 1.

Then, we extracted the information about the animal models. The method of making the animal models for PD was divided into four categories (neurotoxin model, genetic model, neurotoxin and genetic model, and other model groups). The neurotoxin model group includes animal models that are made using any kind of drug injection. Animal models that are made by any gene manipulation were included in the genetic model group. Animal models that were made by injecting any drugs into the animal with gene manipulation were defined as the neurotoxin and genetic model group.

**Figure 2:**
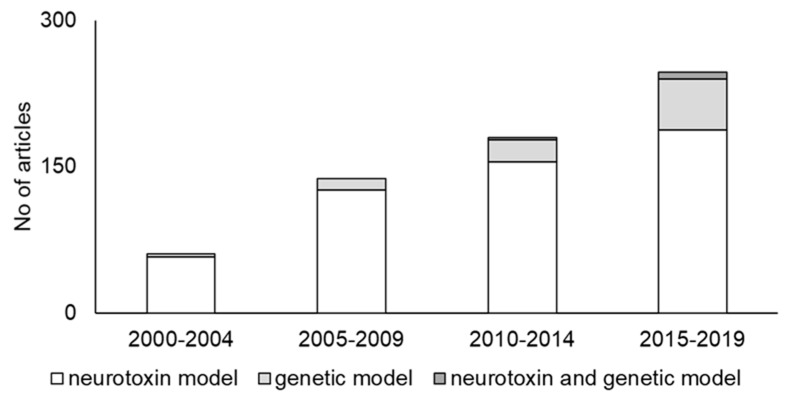
Trends in PD animal models. The number of articles gradually increased.

Thus, we identified 1129 articles, and 626 of these articles were included in the analysis. Reflecting the fact that so many researchers focus on PD, the total number of articles has been increasing (Figure 2). The neurotoxin model group had the largest number of articles and ratio in every time period, while the genetic models have gradually become more common (Figure 2).

### 2.1. Neurotoxin Models

**Figure 3:**
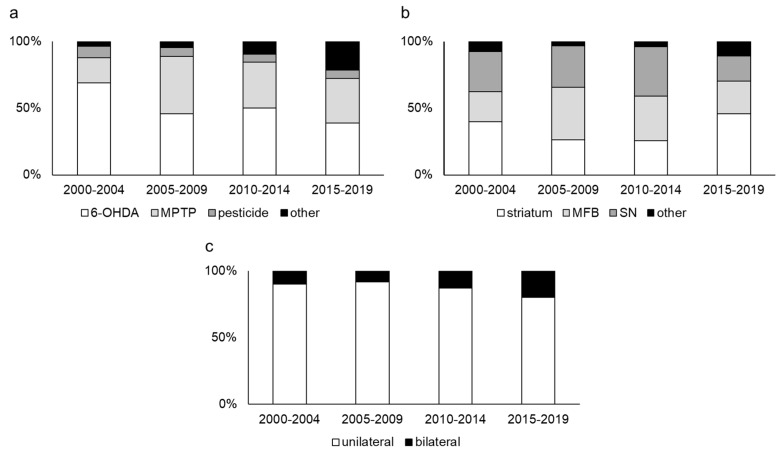
Neurotoxin model results. (**a**) The proportion based on the neurotoxin type. (**b**) The target proportion for 6-OHDA injection. (**c**) The proportion of unilateral or bilateral 6-OHDA injection.

Neurotoxin models are the most classical PD animal models, and they have been the most commonly used model in every time period (Figure 2). Therefore, we can easily compare a new treatment with existing treatments because it has been used to evaluate various therapeutics [11]. In addition, these models can be made relatively easily. These points can be strong advantages and they may explain why the neurotoxin models are the most popular models. Consistent with previously reported speculation [14,15], our research indicated that 6-hydroxydopamine (6-OHDA) and 1-methyl-4-phenyl-1,2,3,6-tetrahydropyridine (MPTP) are the most widely used toxins (Figure 3a).

6-OHDA is well known for its consistent behavioral phenotype from predictable degeneration in dopaminergic neurons [16,17]. Because 6-OHDA does not penetrate the blood brain barrier, direct administration into the brain parenchyma is required. Striatum, medial forebrain bundle (MFB), and SN are common target in all time periods (Figure 3b). 6-OHDA undergoes a rapid non-enzymatic auto-oxidation and produces hydrogen peroxide, superoxide radicals, quinones, and hydroxyl radicals. Although the mechanism is not completely understood, the stress induced by these reactive oxygen species is thought to be caused by 6-OHDA, and this model lacks Lewy-related pathology [18]. 6-OHDA injection into the striatum destroys the axon terminals in the striatum followed by a slowly protracted retrograde degeneration of dopaminergic neurons in the SN [17,19,20]. The symptoms are relatively mild and show a slow progression. However, 6-OHDA injection into the MFB and SN, which are also traditional targets, results in quick and massive dopaminergic cell degeneration. Therefore, the symptoms are quite severe [21]. Although each of these sites may be appropriate for the target of 6-OHDA injection, the rationale for the selected target should be stated clearly.

Unilateral injection was more commonly selected for the 6-OHDA injection (Figure 3c). We also selected unilateral injection because an amphetamine challenge test is available to evaluate motor deficit [22,23,24,25]. However, bilateral injection was performed in some articles. To evaluate spatial memory and recognition, bilateral injections would be valuable [11]. However, it is well known that bilateral lesioning results in aphagia, adipsia, and bilateral motor deficit [26]. The severity of these symptoms depends on the injection site, and sometimes they are life-threatening. Such a highly invasive procedure is appropriate for making PD animal models of PD or not should be more discussed. 

MPTP is one of the most identified neurotoxins that destroys dopaminergic neurons, and it has been commonly used to make animal models for PD research [27]. The severe Parkinsonism in humans that is induced by MPTP has led to the development of this model [28,29]. MPTP easily crosses the blood brain barrier because it is a lipophilic molecule. After systemic administration, MPTP is converted to MPDP+ by monoamine oxidase B in astrocytes. It is then oxidized to MPP+, which is a toxic metabolite that is absorbed by dopaminergic neurons via the dopamine transporter. Absorbed MPP+ disturbs complex-I of the mitochondrial electron transport chain, which results in oxidative stress and a decrease in ATP generation [30,31,32,33]. This is the mechanism by which MPTP degenerates dopaminergic neurons. Although rodents are less sensitive to MPTP toxicity compared to primates, mice are still sensitive. Mice with MPTP treatment are used as a model that is easy to handle, affordable, and highly reproducible by establishing a chronic MPTP protocol [8]. Different from 6-OHDA, α-synuclein may be observed, although this is still controversial [34]. One of the main important limitations in the animal study for PD is the rapid and transient neurodegeneration. However, because MPTP-induced symptoms appear gradually, this limitation may not be applied to this MPTP model.

Some pesticides, such as rotenone, paraquat, maneb, and trichloroethylene, are used as neurotoxins for making PD animal models [35,36,37,38]. Exposure to pesticides is a well-known risk factor for PD [5]. Rotenone readily crosses the brain-blood-barrier and cell membranes because it is lipophilic. Chronic administration of rotenone produces a loss of the dopamine terminals in the striatum followed by degeneration of dopaminergic neurons in the SN [39,40]. However, high mortality, phenotypic variability, and non-PD-related symptoms are reported as disadvantages [41]. Paraquat, which is a weak inhibitor of mitochondrial complex I, is reported to show loss of dopaminergic neurons in the SN [42,43]. Lewy body-like structures have been observed in rodent studies [44,45]. However, we must know that the effects of systematic paraquat administration have been equivocal, and serious doubts were cast by recent studies [46]. Maneb, which is an inhibitor of complex III, is also used as a neurotoxin. The combination of maneb and paraquat is reported to show a significant neural loss in the SN [47]. Trichloroethylene is a neurotoxin that has been used recently. Rodents treated with trichloroethylene show dopamine depletion in the striatum and behavioral deficits [38]. Although various pesticides are used to make PD models like this, the overall proportion of these models is limited (Figure 3a), which may be because there is no obvious advantage of comparisons with other neurotoxins.

Neurotoxin models are valuable because they are easy to handle and compare with previous studies. However, almost none of the neurotoxin models mimic any pathological features of PD, but, rather, they only mimic the symptoms. Further, neurotoxin models reflect the late, chronic, dopamine-depleted state because motor deficits are only visible after severe dopamine depletion [48]. Therefore, for the research to clarify the pathology or to develop a preventative treatment, which is sorely required, neurotoxin models would not be appropriate. Studies with neurotoxin models have been contributing to the development of symptomatic treatment. However, these disadvantages should be kept in mind. One reason for the recent failure to develop new treatments may be the lack of appropriate model use [15]. The neurotoxin models should likely be used as one phenotype of PD.

### 2.2. Genetic Models

Genetic models are a relatively new approach, and they are made by manipulating genes. Transgenic animal models have been generated since the identification of human mutations that are causally linked to familial cases of PD. Various mutations in specific genes that cause mitochondrial dysregulation and energy disturbances are thought to cause PD [49]. Since the first gene, α-synuclein, was found to be unequivocally link to PD, several other PD-linked genes were identified. For example, loss-of-function mutations in Parkin, PTEN-induced putative kinase 1 (PINK1), and DJ-1 are known to be causally linked to familial PD [50,51,52]. The recent advance in transgenic technology has enabled us to model mutations in these genes with knockout rodents. These models may more closely resemble human PD physiology and progression [53,54]. In addition, some models have recently taken advantage either of genes that are known to play a role in familial PD or genes where the expression is significantly changed in PD patients [55]. This suggests that genetic models may be useful to study idiopathic PD, although PD is not thought to be a strongly genetic disease because genetic variations make up only about 5% of all cases [49]. In addition, genetic models allow a new approach in PD research. For example, we can apply treatment before the onset of the symptoms with genetic models [55]. This approach would be valuable in developing a preventative treatment. Our results indicated that the number of genetic models has been increasing, which may be because of this advantage (Figure 2).

α-Synuclein is a small neuronal protein that is particularly located in pre-synaptic terminals [56]. It reportedly has a role in membrane protein regulation and vesicular dynamics [57,58]. The α-syn encoding gene is linked to the main component of the Lewy body, which is observed in the brain od PD patients and also linked to a dominant-type familial PD which is named Park1 [59]. Now, it has become an established fact that mutations in the α-synuclein gene results in dominantly inherited forms of PD [60,61,62]. A variety of mutations have been identified in familial PD [63,64]. Only the duplication or triplication of the α-syn is able to cause PD, suggesting that the expression level of α-syn has a critical role in PD progression [65]. Various types of α-syn transgenic mice have been developed, and many behavioral deficits have been observed. However, no significant nigrostriatal degeneration was identified in most of them [12].

The Parkin gene mutation is known to cause early onset PD. It accounts for half of all PD cases with an onset before 30 years of age and is seldom found in PD with an onset of symptoms after 30 years of age [51,66]. It may also play a role in sporadic PD [67,68,69]. Over 200 Parkin gene mutations have shown to cause PD; they have a recessive mode of inheritance and almost 100% penetrance in homozygous or compound heterozygous individuals [70,71]. Recently, the mechanism by which the Parkin gene mutation causes PD has begun to be revealed. For example, dysfunction in this gene’s activity was reported to cause impaired mitophagy, protein accumulation, and mitochondrial dysfunction [72]. In addition, Parkin and PINK1 function in the same pathway and PINK1 may regulate its interaction and ubiquitination by Parkin. The Parkin knockout animal is a classic genetic PD model that is used by many researchers to study PD. Recently, Parkin activity regulation was reported to be a potential therapeutic strategy. An increase in Parkin activity showed an increase in the mitochondrial quality in Parkin knockout mice [73]. Overexpression of Parkin protected SN from 6-OHDA or α-synuclein overexpression [74,75,76,77]. These findings may represent a new treatment strategy for PD, and this kind of study would be a strength of research using genetic models.

PINK1 is a mitochondrial serine/threonine protein kinase that is intimately involved in mitochondrial quality control. About 4% to 9% of early onset PD in the Asian population and 2% to 4% in the Caucasian population can be accounted by the PINK1 mutation [70]. In vitro studies have suggested that PINK1 works as a sensor of mitochondria with a low membrane potential and activate Parkin to promote sequestration and degradation of defective mitochondria [78,79]. Mutations in PINK1 are reported to link to autosomal recessive PD [50,80,81]. Several PINK1 knockout mice have been used as PD animal models. To the best of our knowledge, none of the PINK1 knockout models show Lewy-related pathology. This feature is consistent with autopsy results from PD patients with the PINK1 mutation, which have shown only mild Lewy-related pathology [82]. In addition, PINK1 knockout mice have been reported to show olfactory and gait disturbances, which is similar to the prodromal symptoms of human PD patients [83]. Therefore, the PINK1 knockout mouse may be a model for prodromal PD [49,84].

The DJ-1 mutation has been linked to autosomal recessive PD [52]. DJ-1 knockout mice show mild motor deficits without dopaminergic neuron loss [85,86]. Some articles indicate that this gene mutation may be involved in sensing or protecting against oxidative stress, although the exact function remains unclear [87,88]. Recently, DJ-1 knockout rats have been used for basic research [13]. In contrast to DJ-1 knockout mice, DJ-1 knockout rats show significant nigral dopaminergic neuron loss that is accompanied by motor deficits [89]. Although there are few studies using DJ-1 knockout rats as a PD model, such PD-like abnormalities would be valuable to study familial PD.

In addition, various gene mutations have been identified and used to develop genetic models. Virus vector injection also enables local gene manipulation. An advantage of the viral vector is the relative ease with which multiple genes or different variants of the same gene can be overexpressed [13]. Comparisons can be made by injecting the same vector into the different species or different brain regions. Another advantage is that we can evaluate the outcome earlier than in transgenic animals. This technique will facilitate the development of transgenic models.

However, most splicing events have not been conserved between humans and mice [90]. It is not clear if the results that are obtained in animal studies can be applied directly to humans. This point should be kept in mind when the results are interpreted.

### 2.3. Neurotoxin and Genetic Models

Recently, combined models, in which a neurotoxin has been applied to genetically manipulated animals, have begun to be used (Figure 2). These models are based on the fact that some genetic models are more susceptible to neurotoxins. For example, it is well known that MPTP induces more neuronal loss in DJ-1 knockout mice than in wild type mice [88]. Conducting research with this kind of animal model may provide new insight from the point of view of the dual-hit hypothesis [91,92]. In addition, such experiments may be useful in capturing the multifaceted nature of PD and could reveal the function of PD-related gene mutations [93]. We know that no toxin or genetic models completely reproduce the human PD. Environmental factors and genetic vulnerability are thought to play a role in the onset and progression of PD [55]. A neurotoxin and genetic model may be the most promising type of model. Because these types of animal models are relatively new, more research and discussion are required. 

### 2.4. The Type of Animal

**Figure 4:**
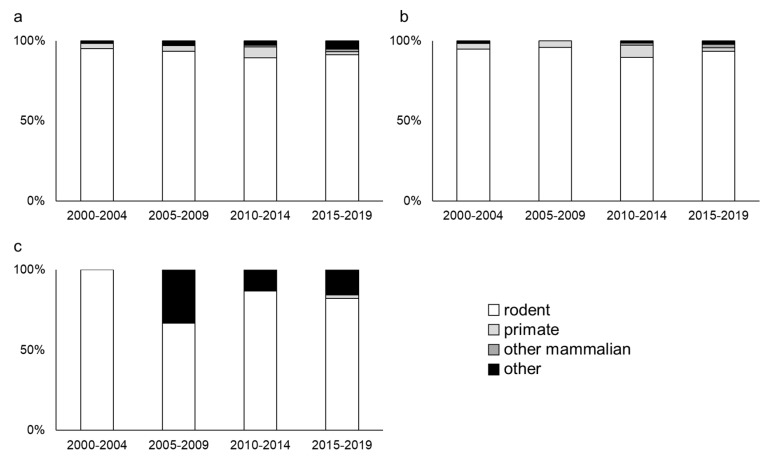
Animal type used for Parkinson’s disease (PD) research. (**a**) The proportion of animal species that are used for research to all types of PD animal models. (**b**) The proportion of animal species that are used for research to neurotoxin models. (**c**) The proportion of the animal species that are used for genetic models.

Various animal species have been used for PD research, and we evaluated the trends in the animal species. The animal species were divided into four categories (rodent, primate, other mammalian, and other groups). The other mammalian group includes animals such as the minipig, dog, and cat. The other group includes animals such as *Caenorhabditis elegans* (*C. elegans*), drosophila, and zebra fish. Rodents have been the most commonly used animal in every time period (Figure 4a). Rodents do not require a special setup for breeding and are easy to handle. Their size is moderately small, and their anatomy is relatively similar to that of humans. Thus, the rodent is thought to be one of the most classical animal species that are used for PD research [94], which is consistent with our results. The animal species that are used in the neurotoxin models show a similar trend. Vast majority of animals used for neurotoxin models are rodents (Figure 4b).

However, different trends can be observed for the genetic models (Figure 4c). The other group is used more often for genetic models. Whole genome information has been sequenced in some organisms such as *C. elegans* and drosophila. In addition, their lifespan is relatively short. Although they do not have intrinsic α-synuclein, such advantage would be valuable for research involving gene manipulation. This may explain why this trend has occurred. 

Primates are often used in PD research, although the number is relatively low. Primates are the animal species that is the closest to humans. However, handling and breeding primates is difficult. In some institutes, using primates for basic research would be difficult. However, primates are useful in PD research, but the cost and ethical concerns may prevent primates from being more widely used. 

## 3. Summary and Future Prospective

**Figure 5:**
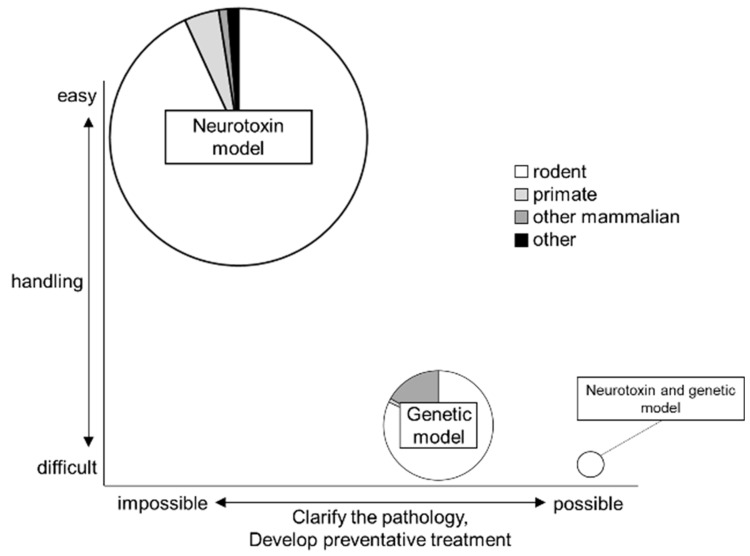
Trends in animal models that are used for PD research. The size of the Venn diagram indicates the number of studies that were conducted using each model since 2000. The neurotoxin models are most commonly used, and they are the easiest animals to handle. However, these rodent models are not appropriate for studies to clarify the pathology or to develop preventative treatment. Although genetic models and neurotoxin and genetic models would be relatively difficult to handle, studies involving these models may be used to clarify the pathology or to develop preventative treatment.

The PD studies mainly use the neurotoxin models, which are easy to handle. The number of genetic models and neurotoxin and genetic models is relatively small, although it has been increasing (Figure 5). The advantages of each model are obvious, and the model that is used can be selected based on the purpose of the research. However, the disadvantages are also obvious. Various difference between human PD and animal PD model have been known (Table 1). It is important to understand both sides to conduct the appropriate research.

What is necessary for PD research? Finding new animal models for PD research would be valuable, and research to develop new models is ongoing. Recently, new techniques have begun to be used in PD research. The CRISPER/CAS9 technique was applied to monkeys to clarify the function of the PINK1 mutation [95]. Halorhodopsin was introduced into the SN of rats to mimic the various stages of PD [96]. Cutting-edge techniques such as CRISPER/CAS9 and optogenetics will advance PD research. However, the validity of these new models requires further investigation.

Except for the above, there are some animal models which can be valuable for the PD research. For example, α-Synuclein propagation model would be one of them. Prion-like propagation of α-Synuclein has been reported by various researchers and animals with the injection of brain homogenates with α-Synuclein used for the researches of the α-Synuclein related diseases [97,98]. These researches are not included in our research because the authors do not clearly state that it is an animal model of Parkinson’s disease in the article. However, there is no doubt for the value of these animals for the PD research as PD is one of α-Synuclein related diseases. At the same time, it can be a drawback of our study.

Accumulation of knowledge about the existing models is also important. For example, some biomarkers were reported to change in the MPTP mouse, which is similar to PD patients [99]. This result may support the validity of the MPTP mouse model. This kind of “back translation research”, which connects clinical research and animal research to consolidate the validity of animal models, is valuable. In addition, comparing the various PD models may also be helpful to interpret the results. Some researchers have begun to use multiple models in one study [1,93,94,100,101,102]. Studies to clarify the pathology or to develop new treatment would be the main focus of these studies. However, we should not miss the importance of these kinds of studies because no animal model can completely mimic human PD.

## 4. Conclusions

Many methods have been developed and various animal species are used for PD animal models. Rodent models made using neurotoxin are most commonly used as PD animal models, and genetic models have been increasing in popularity. We believe that almost every PD model is essential for PD research as long as the appropriate model is selected for the hypothesis. Because it is impossible to replicate human PD completely in animals, we should keep paying attention to the validity of the animal models and the results from the experiments with these animal models. 

## Figures and Tables

**Table 1:** The difference between human PD and animal PD model.

Animal Model	The Main Difference Between Human PD and Animal PD Model	
**Neurotoxin model**	6-OHDA	Rapid progression. No lewy related pathology. The pathology is completely different.
	MPTP	Movement disorder is not obvious. The pathology is completely different.
	Pesticides	Rapid progression. The pathology is completely different.
**Genetic model**	Movement disorder is relatively rare. Lewy related pathology can be identified in a few models. Dopaminergic neuronal damage is relatively rare.	

## Abbreviations

PD	Parkinson’s disease
SN	substantia nigra
6-OHDA	6-hydroxydopamine
MPTP	1-methyl-4-phenyl-1,2,3,6-tetrahydropyridine
MFB	Medial forebrain bundle
PINK1	PTEN-induced putative kinase 1
*C. elegans*	*Caenorhabditis elegans*
